# Acoustic, Pitfall Trap, and Visual Surveys of Stored Product Insect Pests in Kenyan Warehouses

**DOI:** 10.3390/insects10040105

**Published:** 2019-04-12

**Authors:** Anastasia Njoroge, Hippolyte Affognon, Uwe Richter, Oliver Hensel, Barukh Rohde, Davie Chen, Richard Mankin

**Affiliations:** 1 Department of Entomology, Purdue University, West Lafayette, IN 47907, USA; annwanjiru608@gmail.com; 2 Conseil Quest et Centre Africain pour la Recherche et le Développement Agricoles (CORAF), 7 Avenue Bourguiba, Dakar, BP 48, Dakar RP, Senegal; H.Affognon@coraf.org; 3 Faculty of Agricultural Engineering, University of Kassel, D-37213 Witzenhausen, Germany; judge@uni-kassel.de (U.R.); agrartechnik@uni-Kassel.de (O.H.); 4 Department of Electrical and Computer Engineering, University of Florida, Gainesville, FL 32611, USA; barukh94-work@yahoo.com; 5 US Department of Agriculture, Agricultural Research Service, Center for Medical, Agricultural and Veterinary Entomology, Gainesville, FL 32608, USA; chen.davie@husky.neu.edu

**Keywords:** *Prostephanus truncatus*, *Sitophilus zeamais*, *Tribolium castaneum*, *Sitotroga cerealella*, postharvest loss, grain, pest, background noise

## Abstract

Grain production is an important component of food security in Kenya but due to environmental conditions that favor rapid growth of insect populations, farmers and other agricultural stakeholders face ongoing and novel challenges from crop and stored product pest insects. To assist development of methods to reduce economic losses from stored product insect pests in Kenya, acoustic, visual, and pitfall trap surveys were conducted in five grain storage warehouses. Two commercially available acoustic systems successfully detected the pests of greatest economic importance, *Sitophilus zeamais* (Motschulsky) and *Prostephanus truncatus* (Horn). Other insects of lesser economic importance also were observed in the visual surveys, including *Sitotroga cerealella* (Olivier) (Lepidoptera: Gelechiidae), and *Tribolium castaneum* (Herbst). This study demonstrated that the use of acoustic technology with visual surveys and pitfall traps can help managers to identify and target infestations within their warehouses, enabling them to reduce postharvest losses. With most warehouses being located in relatively noisy urban or peri-urban areas, background noise considerations are being incorporated into the design of future acoustic detectors for stored pest infestations. Kenya must import grain yearly to meet consumption needs; however, if the current yearly postharvest losses of 20–30% in warehouses decreased, import costs could be reduced considerably.

## 1. Introduction

Over $4 billion USD in food losses occur yearly in Africa due to inefficiencies in the chain of production, storage, and marketing activities that connects farmers to consumers [1]. Kenya has developed a Strategic Grain Reserve to store sufficient grain for release into markets if supplies fall below typical levels of consumption [2]. The government purchases backup maize yearly that can be released in an emergency. There is wide recognition that strategic grain reserves play a vital role in ensuring Kenyan food security. Additionally, it is anticipated that the recent invasion of *Spodoptera frugiperda* (J.E. Smith) (Lepidoptera: Noctuidae) [fall armyworm] into sub-Saharan Africa will lead to 20–50% maize yield loss [3], further increasing the need for imports to bolster backup maize supplies.

Kenya currently experiences an estimated 20–30% postharvest loss of staple grains yearly, which poses great challenges to the country’s food security and economic development [4]. *Prostephanus truncatus* (Horn) (Coleoptera: Bostrichidae) [larger grain borer], *Sitophilus zeamais* (Motschulsky) (Coleoptera: Curculionidae) [maize weevil], *Tribolium castaneum* (Herbst) (Coleoptera: Tenebrionidae) [red flour beetle] and *Sitotroga cerealella* (Olivier) (Lepidoptera: Gelechiidae) [Angoumois grain moth] are the major maize pests in sub-Saharan Africa [5]. Postharvest losses significantly endanger the livelihoods of stakeholders across the value chain by reducing income and profitability, and given that overall production in sub-Saharan Africa is increasing while the percentage postharvest loss remains unchanged, the nature and extent of such losses is coming under increased scrutiny [6].

Managers of bulk grain storage facilities fumigate with phosphine gas routinely; however, *Rhyzopertha dominica* (Fabricius) (Coleoptera: Bostrichidae) [lesser grain borer], *T. castaneum,* and possibly other postharvest pests have been developing resistance to phosphine [7]. In addition, gas tightness is not complete in many warehouses and fumigation needs to be augmented with additional management. Routine monitoring and timely inspection for pests facilitates alternative treatment of infestations before they cause economic damage. Commonly used monitoring methods include visual inspections in and around warehouses, examination of grain samples, measurements of temperature changes in bulk grain, and widespread placement/inspection of insect traps [8]. Visual examination and insect traps unfortunately cannot detect larvae hidden inside the grain kernels until they finish development and emerge.

Acoustic technology can detect both adult and hidden larval infestations [9,10,11], providing estimates of population density [12] and spatial distribution [13,14] to warehouse managers who time and target grain management efforts. Also, acoustic technology can be used to test the efficacy of other control treatments such as hermetic storage [15,16]. Recently, significant effort has been directed towards integrating acoustic technology into grain storage management in Africa [17,18,19]. Several grain storage sites near Nairobi were visited to gain consent for acoustic surveys of hidden stages and adult insects within their premises and consider the feasibility of incorporating acoustic technology into Kenyan postharvest pest management programs. This report presents the results of a survey conducted using two commercially available acoustic sensor systems concurrently with commonly used pitfall traps at five sites where consent was obtained. It was hypothesized that (i) detection of adult and larval infestations would be possible in the presence of background noise, (ii) data from two different measurement platforms would be comparable, and (iii) pitfall trap catch data would correlate with acoustic signals recorded.

## 2. Materials and Methods

### 2.1. Recording Sites

A preliminary survey was conducted in March 2016 to assess background noises in the vicinity of four warehouses located in urban and peri-urban settings (Nairobi and Thika) as well as rural settings (Embu and Ishiara). Previous acoustic surveys in field and warehouse environments [9] suggest that, at a very early stage in the planning of acoustic sensor installation in commercial and strategic warehouses for grain storage, it is necessary to identify sources of background noise and mitigate their effects when possible. Recording equipment was set up and left overnight for 24 h recordings to be taken in each location. Aural screening of the recordings confirmed that background noise levels were generally higher at Nairobi and Thika than at Embu and Ishiara, but sufficient periods of low background noise occurred at all locations to enable acoustic surveys in the warehouses.

**Figure 1:**
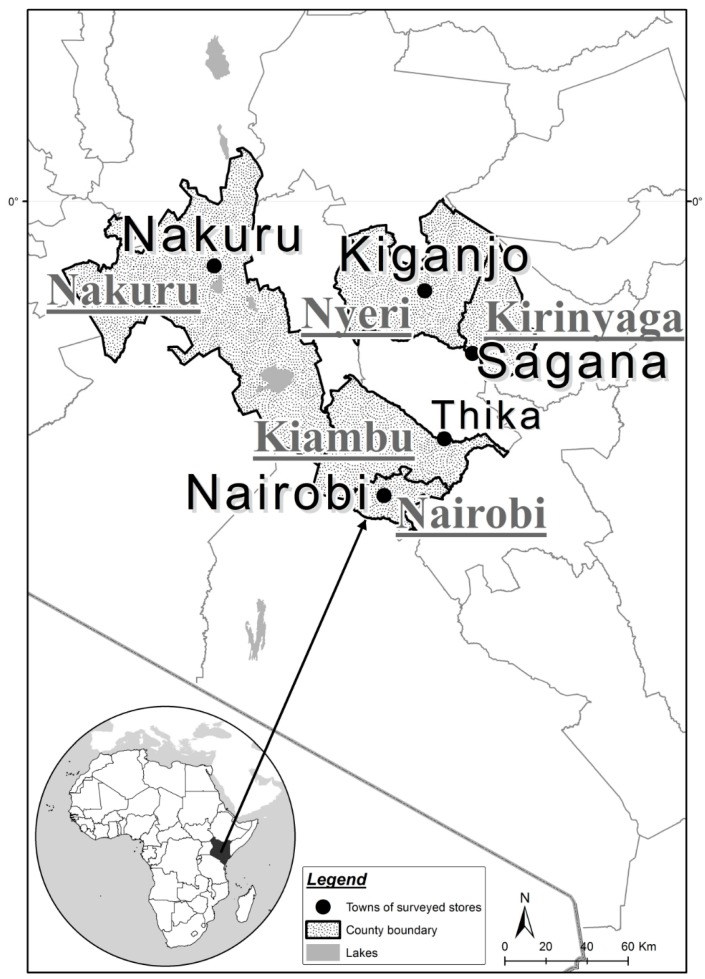
Locations of maize warehouses acoustically surveyed in Kenya. Names of counties are underlined.

In June 2016, insect sound recordings were collected from 50 kg bags of maize at warehouses in five separate counties: Nairobi, Kiambu, Kirinyaga, Nyeri, and Nakuru, as shown in Figure 1. The sites had similar, subtropical highland climatic conditions with moderate daily temperatures, varying slightly with altitude: Thika, 1631 m; Sagana; 1762 m, Nairobi, 1795 m; Nakuru 1850 m; and Kiganjo, 2161 m. Their average temperatures in June are 21.3 °C, 22.4 °C, 19.0 °C, 18.8 °C, and 17.5 °C, respectively. Each warehouse had several stacks of maize bags under routine fumigation, most of which nevertheless contained various postharvest pest species at different infestation levels.

### 2.2. Sampling Methods

Visual inspection for infestation was conducted to identify likely infested stacks which, according to the recommendations of ISO Standard 6322-1 [20], typically were along the stack edges, at the top of the bulk, and areas with spillages. After identifying a stack likely to support infestation, 12 bags were drawn randomly from the surface of the stack, brought to the floor, and set vertically for acoustic recordings and pitfall trap insertion, except for several high stacks at Thika and Kiganjo, where the acoustic system was brought to the top of the stack for signal collection.

### 2.3. Insect Trapping

For each of the 12 selected bags per warehouse, non-pheromone Storgard WB Probe II traps (pitfall traps) were set up prior to acoustic recording to collect samples of free-moving insects in the grain. The grain probe trap excludes grain kernels but permits insect entry through its perforated walls after which the insects fall into the collecting vial from which they cannot escape. All traps were labelled as well as the plastic containers in which the trap contents were emptied. The traps were retrieved 2–3 h later, at the end of the acoustic recordings, and the contents were taken to the laboratory where they were poured into vials and sealed. After surveys were completed, the insects were identified to species level and counted.

The most frequently encountered insects were distinguished based on their morphological features [21]. *Sitophilus zeamais* was identified by its 2.5–4.4 mm long rostrum and dark brown color, sometimes with four lighter spots on the wing cases [18]. *Prostephanus truncatus* was recognized by the position of its head, “tucked” under the thorax so that it is invisible from above, and the prominent pattern of tubercles on the thorax [18]. *Tribolium castaneum* was identified as an elongated reddish-brown beetle [8]. Other less frequently encountered insects that did not match these three were counted as “other species”. Larvae were not identified to species level and were reported as “mixed larvae”. Means and standard errors of the counts in different categories in the 12 bags from each site were subjected to analysis of variance.

### 2.4. Recording Equipment and Set-Up

The recordings were collected using two different acoustic systems, hereafter designated *IMC* and *AEC*, enabling direct comparison of the detection ranges and background noise discrimination capabilities of each system in Kenyan warehouse environments. The *IMC* system included a 0.5″ microphone (Model 378B02, PCB Piezotronics Inc., New York, NY, USA) attached to a preamplifier system (imc C-SERIES, CS-3008-N, imc Meßsysteme GmbH, Frankfurt, Germany), as described in [18,19]. The *AEC* system included a 16 cm length × 6 mm diam stainless steel probe attached to a sensor–preamplifier module (model SP-1L, Acoustic Emission Consulting [AEC] Inc., Sacramento, CA, USA) connected to an amplifier (AED-2010, AEC Inc. Sacramento, CA, USA), leading to a digital audio recorder (model HD-P2, Tascam, Montebello, CA, USA) which stored signals at a 44.1 kHz digitization rate, as described in [17,22]. Records of 3–5 min each were collected over a 5 day period from a total of 60 different bags.

Weather conditions were dry with no rain or wind present throughout the survey periods. Each site was unique, with Kiganjo and Sagana located in relatively quiet environments and Nairobi, Nakuru, and Thika in urban or peri-urban environments characterized by intermittently high levels of background noise. Other sources of noise included birds singing, vehicle movement and beeping, on-site machine noises, and worker activity. Monitoring was conducted with headphones before each recording to help identify times when background noise levels greatly exceeded the insect sound pressure levels, in which cases recording was postponed. Nevertheless, recordings were rarely totally free of nontarget background noise, and automated signal processing was conducted to discriminate the targeted insect signals from the noise (see below). Testing began at approximately 10:00 a.m. and typically continued for about 3 h. For each bag, recordings were made simultaneously with both systems except at the Thika warehouse when a several-hour power failure precluded data collection by the *IMC*, which required a standard line connection.

### 2.5. Automated Classification of Insect Sounds and Background Noise Signals

Signals from the *IMC* system were converted from .ccv (curve configuration files) to .wav (wave audio files) format using a custom program written in MATLAB Release 2012b (The MathWorks Inc., Natick, MA, USA). The *AEC* signals already were in .wav format. The recordings were band-pass filtered between the 1–10 kHz range of greatest insect sound amplitude [9] and pre-screened using Raven Pro 1.5 Beta Version software [23] (Cornell Lab of Ornithology, New York, NY, USA; Charif et al., 2008). Prescreening entailed playback, oscillogram, and spectrogram analysis of each file to locate periods of insect sound impulses and discard periods of loud background noises.

To discriminate insect sounds from background noise, we analyzed the signals using DAVIS signal processing algorithms [9,24]. Preliminary screening of initially collected files indicated that the *IMC* microphone and the AEC sensor had different patterns of spectral sensitivity; consequently, the mean spectra (profiles) [9] of insect sounds were different for each system and separately constructed, system-specific profiles were applied by the DAVIS algorithms to identify and discriminate the insect sounds from background noise. Two spectral profiles were constructed from frequently occurring insect sounds recorded by each system. For *AEC* recordings, one profile was collected as a mean spectrum of 139 consecutive impulses recorded over a 62 s interval from the Kiganjo warehouse. The second profile was constructed as a mean spectrum of 33 consecutive impulses recorded over a 20 s period from a bag at the Nakuru warehouse. These same *AEC* profiles were used successfully as well in the study by [18]. For *IMC* recordings, the first profile was constructed as a mean spectrum of 26 impulses collected over a 26 s period from a bag at Kiganjo. The second was constructed from 61 impulses collected over a 42 s period, also from a bag at Kiganjo. In addition, prescreening identified bird noise that occurred frequently in all the warehouses surveyed, and therefore a bird profile was calculated to facilitate discrimination between insect sound impulses and background noise, as described in [25]. The *IMC* and *AEC* bird profiles were obtained from recordings at Kiganjo. The warehouse at Nairobi also had considerable numbers of recordings with bird noise.

The sound impulses in each *IMC* or *AEC* recording were least-squares matched by DAVIS against the three corresponding insect and bird profiles above and were assigned to the profile type of best fit as in [9]. Impulses classified as bird or other background noise were discarded. DAVIS classified impulse trains containing >2 and <200 impulses that matched the two insect sound profiles as insect sound bursts in each recording, based on the high likelihood that they were produced by insects and not by background sounds [9,24]. The discrimination was based on the fact that insect movement and feeding activity generates distinctive trains (groups) of 1–30 ms impulses that only rarely occur as features in background noise [9,26].

The times of occurrence and profile type of each insect sound burst were saved in a spreadsheet for statistical analyses. Three parameters of quantifying different aspects of acoustic activity were calculated for signals classified under the two insect sound profile types: rate of bursts, *R_b_*_,_ with units of number of bursts/s (which is a measure of the frequency of occurrence of individual insect movements); counts of impulses per burst, *N_b_* (indirectly quantifying the duration of individual insect movements); and rates of burst impulses, *R_bimp_* (number of impulses detected only within bursts, divided by the recording duration in s), which is a measure of the total insect effort as described in [18,19]. Overall measures for the acoustic parameters for each recording were calculated as the sums of the values obtained for each insect sound profile, as in [18,19].

## 3. Results

### 3.1. Pitfall Trap Counts and Visual Surveys

Trap capture data is shown in Table 1 with assessments of mean captures across species and warehouses. *P. truncatus* and *S. zeamais* were present in four out of five, while *T. castaneum* was present in all five warehouses surveyed. Other species and mixed larvae were observed in three and four sites, respectively. It is notable that *T. castaneum* were found in high numbers at all five sites. The highest mean counts recorded per site were obtained for *P. truncatus* in Kiganjo at 39.08, and *T. castaneum* in Nairobi, Nakuru, Sagana, and Thika at 39.42, 27.11, 5.86, and 12.50, respectively. Also of interest was the presence of large numbers of *S. cerealella* larvae at Thika. They were not captured in the traps, as they were mostly on walls, floors, and on top of bags with some also dropping from the ceiling.

Significant differences were observed in the species counts across warehouses, as shown in Table 1. For *P. truncatus* and other species there was little variation in Nairobi, Nakuru, Sagana, and Thika. The biggest variation was observed for *T. castaneum,* but other species also showed considerable variation across warehouses. Overall, Sagana had the lowest infestation levels while Kiganjo had the highest levels.

### 3.2. Acoustic Assessment of Infestation

Means ± standard error of mean (SEM) of three acoustic parameters of insect activity in different warehouses are shown in Table 2, based on analyses of variance across locations, as shown in Table 3, and Student’s *t* test analyses of means obtained by *AEC* and *IMC* acoustic systems, as shown in Table 4. It should be noted that we attempted to redo recordings when background noise occurred for long periods in recordings, but it was not always feasible to do so, and complete pairs of *AEC* and *IMC* recordings were obtained only for eight bags at Kiganjo, seven at Nairobi, six at Sagana, and five at Nakuru.

Because the infestation levels in different bags varied considerably within warehouses, no significant differences were found across warehouses in rates of bursts, impulses per burst, or rates of impulses within bursts compared separately for *AEC* and *IMC* systems, as shown in Table 3. Only one significant difference (*p* > 0.05) was observed between *AEC* and *IMC* measurements in any warehouse—a comparison between the rates of bursts, *R_b_*, detected by *AEC* and *IMC* systems at the Kiganjo warehouse, as shown in Table 2 and Table 4. When bags were pooled across warehouses, the values of *R_b_* measured across bags were proportional to the numbers of insects later recovered from the bags, as reported in the next section.

### 3.3. Relationship between Burst Rates and Counts of Insects Recovered from Bags

Sound impulses matching insect spectral profiles were detected in all recordings at each site and all bags tested were rated at medium or high likelihood of infestation based on the total rates of insect sound bursts exceeding a detection threshold of 0.02 burst/s [24]. It was of interest to sum the trap counts of the two most important pests, *P. truncatus* and *S. zeamais*, as a single total, *T_c_*, given that the acoustic signals of these two insects were not readily distinguishable. Previous studies [9] suggested that the insect sound burst rates, *R_b_*, from each of the systems, *AEC* and *IMC*, would be approximately proportional to *T_c_*, i.e., the statistical model would be:

*R_b_* = *T_c_*. (1)

In addition, the *AEC* and *IMC* systems were expected to detect sound bursts at different rates within maize bags due to differences in the positions of the insects relative to the detectors as well as differences in the range of detection. The *IMC* detected insects over approximately 25 cm distances from the top of the bag, while the *AEC* collected signals along the whole 16 cm length of the probe [17]. On average, however, randomizing over position between sensor and insect, the insect sound burst rate detected by the *IMC* system, imc*R_b_*, was expected to be proportional to the rate detected by the *AEC* system, aec*R_b_*, with the statistical model:

imc*R_b_* = aec*R_b_*. (2)

**Figure 2:**
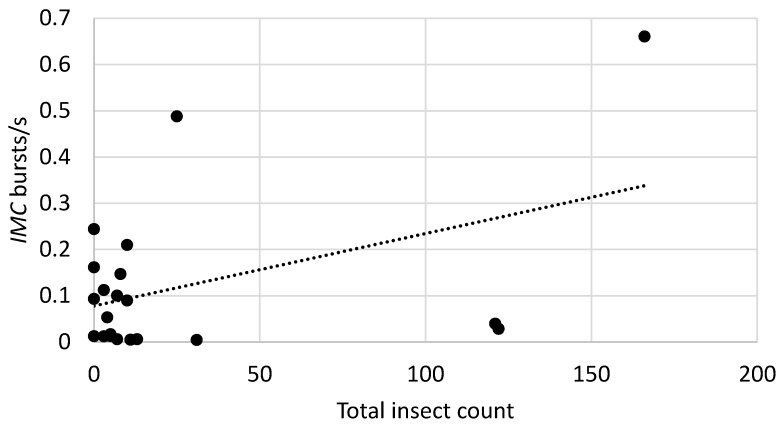
Relationship between insect sound burst rates and total counts of *P. truncatus* and *S. zeamais* for the *IMC* system in recordings from 21 bags at Kiganjo, Nairobi, Nakuru, and Sagana warehouses. Filled circles indicate burst rates from individual bags with specified total counts of the two species. Dotted line indicates the linear regression of insect sound bursts/s on the total counts.

**Figure 3:**
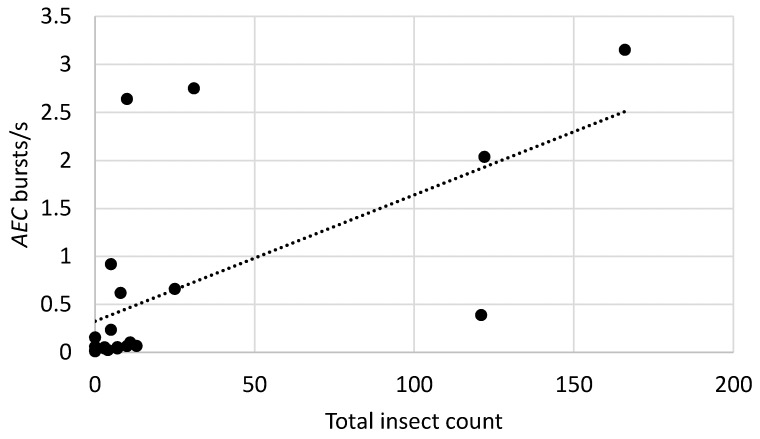
Relationship between insect sound burst rates and total counts of *P. truncatus* and *S. zeamais* for the *AEC* system in recordings from 21 bags at Kiganjo, Nairobi, Nakuru, and Sagana warehouses. Filled circles indicate burst rates from individual bags with specified total counts of the two species. Dotted line indicates the linear regression of insect sound bursts/s on the total counts.

**Figure 4:**
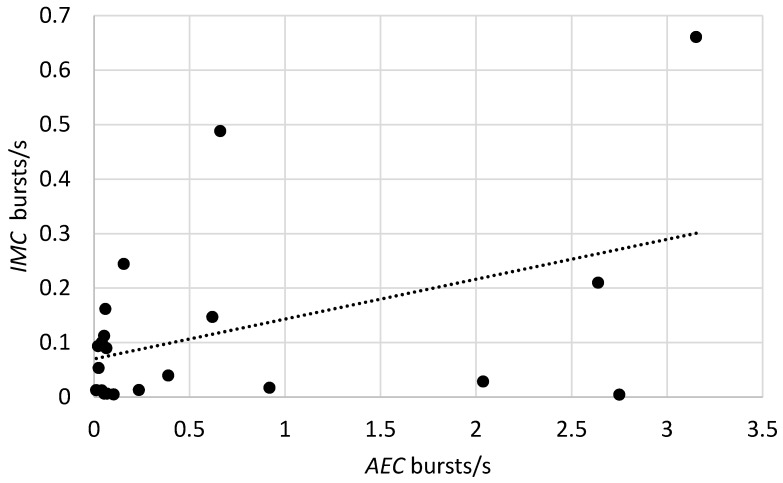
Comparison of insect sound burst rates from 21 bags at the Kiganjo, Nairobi, Nakuru, and Sagana warehouses in which recordings were obtained from both the *IMC* and *AEC* acoustic system. Filled circles indicate *IMC* system burst rates from individual bags with specified *AEC* system burst rates. Dotted line indicates the linear regression of *IMC* rates on *AEC* rates.

The models were tested for insect sound burst rates from 21 bags at the Kiganjo, Nairobi, Nakuru, and Sagana warehouses in which recordings were obtained from both *AEC* and *IMC* systems. The slopes of the regressions were statistically significant for each of the models, as shown in Table 5, and the regression lines are shown in Figure 2, Figure 3 and Figure 4.

## 4. Discussion

Previously reported [1] as well as predicted [3] economic losses from crop and stored product insect pests in Kenya and surrounding regions indicate concern for the high magnitude of current and future losses associated with storage of staple cereals in sub-Saharan Africa. The survey results concur with such concerns, reporting infestation of at least two species of postharvest insect pests in all warehouses surveyed. Visual inspection of storage bags provided a subjective impression while the pitfall traps and acoustic surveillance helped quantify estimates of infestation in the warehouses at the time of survey.

Pitfall trap counts provide sampling information about local insect populations that can be useful for pest management programs [8]. Two of the species found in the traps, *P. truncatus* and *S. zeamais,* are of great economic significance in maize storage in sub-Saharan Africa. Their presence in bulk grain storage poses a threat to the food security of the populations depending on that grain for survival. Other examples of significant numbers of internally feeding pests observed in the pitfall traps included *S. oryzae*, *S. granarius*, *R. dominica*, and several bruchids noted also in [8]. Though *T. castaneum* levels were high in all warehouses surveyed, it was not considered a serious pest because it is an external feeding species amongst others such as *Plodia interpunctella* (Hübner) (Lepidoptera: Pyralidae) [Indian meal moth] and *Oryzaephilus surinamensis* (Linnaeus) (Coleoptera, Silvanidae) [sawtoothed grain beetle].

The results of the visual and acoustic assessments of infestation of the 50 kg storage bags suggest that both the *AEC* and *IMC* systems provided consistent quantitative measurements. Previous research has shown, however, that among the commercially available detection systems, piezoelectric sensors have greater sensitivity to insect-produced sounds because the signals encounter less attenuation as signals traverse from the insects to the sensors across different media [27]. In addition, skill and experience were needed to identify periods when background noise was low enough for automated discrimination of insect sounds from background noise.

Proximity of the insects to the sensors is known to be another important factor contributing to the sensitivity of an acoustic system to detect insect infestation. The use of waveguide probes can improve the detection range by increasing the volume of grain close enough to the sensor for detection, thus improving the accuracy of detection. In this study, the rates of insect sound bursts detected and the rate of burst impulses, were consistently greater for the *AEC* probe system than for the *IMC* system. Such results were in agreement with Leblanc et al. [10], who compared acoustic data collected with a 4 cm diam, 1.4 m long field probe (Early Warning Diagnosis (EWD) P3™, Systelia Technol., Hyéres, France) with acoustic data from a short probe for kg-sized samples, EWD LAB™. The sensitivity of the LAB system was lower than that of the P3 system due to the difference in detection range.

To improve the sensitivity and accuracy of detection in the presence of background noise, efforts have been directed towards constructing sound-attenuating boxes lined with foam and fitted with piezoelectric sensors, an example of which is shown in [28]. These innovations are of interest for African agriculture because the sound-attenuator boxes can be fabricated from locally available material. A sound-attenuation box coupled with prototype sensors such as those of [17], can be used for low-cost insect detection in African grain stores. This will complement prototypes and sensors developed previously, e.g., the EWD [10].

The statistically significant correspondence between the magnitude of insect sound burst rates and counts of insects captured in the probe traps confirms that acoustic detection is a useful tool for detecting and monitoring infestations in Kenyan grain warehouses. An important benefit of acoustic systems is their identification of hidden as well as visible infestations of important *P. truncatus* and *S. zeamais* pest species, which provides earlier detection. However, the effects of distance between insect and sensor on detectability noted above, as well as the considerable variation of an insect’s level of activity over time [9], and the effects of temperature on insect activity [29], combine to reduce the precision of any single acoustic measurement of insect activity, which explains much of the variability seen in the relationships between sound burst rate and insect counts in Figure 2 and Figure 3. Nevertheless, the use of acoustic indicators to estimate infestation likelihood [9,24] in individual bags can provide guidance to warehouse managers in targeting infestations. Acoustic methods can readily complement other efforts to reduce economic and qualitative losses in warehouses in Africa, including the use of modified atmospheres [30] and hermetic storage bag technology [31].

It is worth noting that several cell phone sound detection apps have been developed, e.g., [32], some of which already have been adapted for insect detection [33]. There is potential that these or similar apps can be modified or further developed for stored product insect detection by incorporation of the DAVIS [9] signal analysis algorithms [34]. Microphone or piezoelectric sensor systems can be coupled with the smart phones and the app creating a useful tool for scouting for insects in grain stores. In addition, there is the potential that sensor output can be routed to WIFI and, instead of being used only in scouting programs, they could be placed at strategic locations in the warehouse and programmed to text the warehouse managers when acoustic indices register a high likelihood of insect infestation.

## 5. Conclusions

Experience gained from the Kenyan stored product insect acoustic detection study indicates that acoustic, visual, and pitfall trap surveys all contribute information useful for early detection and management of visible and hidden pest infestations but suggests that challenges remain in designing user-friendly acoustic systems that automatically discriminate out background noise often present in warehouses. Efforts are in progress to incorporate additional spectral and temporal pattern features of sounds produced by target insects into cost-effective acoustic detection systems. Knowledge of early infestation can assist warehouse managers in maintaining strategic grain reserves with scarce resources. Improved monitoring combined with innovations such as hermetic storage bags may enable reduced reliance on grain imports.

## Figures and Tables

**Table 1:** Analysis of counts of insects captured per 50 kg bag in each warehouse (mean ± standard error of mean [SEM]). Means for different species in the same warehouse that are followed by the same (small) letter are not significantly different from each other (*p* > 0.05). Means of the same species in different warehouses that are followed by the same (capital) letter are not significantly different from each other (*p* > 0.05). Means were separated using the Bonferroni adjustment.

Insect Category	Location				
	Kiganjo	Nairobi	Nakuru	Sagana	Thika
*Prostephanus truncatus*	37.69 ± 13.80aA	0.25 ± 0.25aB	0.89 ± 0.54aB	0.57 ± 0.42aB	0.00 ± 0.00aB
*Sitophilus zeamais*	6.23 ± 5.82bA	20.42 ± 6.71bB	24.56 ± 8.70bB	0.00 ± 0.00aC	0.25 ± 0.25aC
*Tribolium castaneum*	39.08 ± 7.37aA	39.42 ± 4.75cA	27.11 ± 7.64bB	5.86 ± 1.74bC	12.50 ± 1.84bD
Other	4.07 ± 0.78bA	0.08 ± 0.08aB	0.56 ± 0.44aB	0.00 ± 0.00aB	0.00 ± 0.00aB
Mixed larvae	7.54 ± 1.75bA	0.00 ± 0.00aB	3.67 ± 1.09aC	0.14 ± 0.14aB	0.42 ± 0.42aB

**Table 2:** Mean comparisons of burst rate, *R_b_*, impulses per burst, *N_b_*, and rates of impulses in bursts, *R_bimp_*, recorded by the *AEC* and *IMC* systems in bags at different warehouses. Lack of line current precluded use of the *IMC* system at Thika. Means of a given acoustic parameter obtained with different acoustic systems at the same warehouse that are followed by a different letter are significantly different from each other (*p* > 0.05) under the Tukey–Kramer honest significant difference (HSD) test.

Location	Mean ± SEM of Acoustic Parameter Measured by *AEC* or *IMC* System					
	*R_b_*		*N_b_*		*R_bimp_*	
	*AEC*	*IMC*	*AEC*	*IMC*	*AEC*	*IMC*
Kiganjo	1.204a ± 0.432	0.209b ± 0.085	11.52 ± 3.96	41.63 ± 19.86	15.32 ± 7.13	6.87 ± 4.29
Nairobi	0.581 ± 0.381	0.0446 ± 0.0170	4.84 ± 0.42	20.27 ± 11.43	2.91 ± 1.96	0.777 ± 0.367
Nakuru	0.358 ± 0.121	0.125 ± 0.056	32.26 ± 13.78	9.79 ± 3.45	15.18 ± 7.67	1.85 ± 1.02
Thika	0.200 ± 0.067	-	43.60 ± 22.94	*-*	7.23 ± 3.77	
Sagana	0.093 ± 0.034	0.094 ± 0.038	35.44 ± 13.24	11.41 ± 8.45	5.38 ± 3.26	1.28 ± 0.64

**Table 3:** One-way analysis of variance of acoustic parameter means across locations.

Acoustic Parameter	Df (Parameter, Error)	*F*	*p*
*AEC R_b_*	4, 27	2.22	0.094
*IMC R_b_*	3, 22	1.50	0.242
*AEC N_b_*	4, 27	1.90	0.139
*IMC N_b_*	3, 22	1.17	0.343
*AEC R_bimp_*	4, 27	1.18	0.340
*IMC R_bimp_*	3, 22	1.22	0.325

**Table 4:** Student’s *t* test comparisons of differences between means of *AEC* and *IMC* acoustic parameters at different locations.

Acoustic Parameter	Df	*t*	*p*
Kiganjo *R_b_*	8	2.26	0.028 *
Nakuru *R_b_*	5	1.75	0.117
Nairobi *R_b_*	7	1.40	0.210
Sagana *R_b_*	6	0.008	0.992
Kiganjo *N_b_*	8	1.48	0.178
Nakuru *N_b_*	5	1.58	0.181
Nairobi *N_b_*	7	1.35	0.226
Sagana *N_b_*	6	1.75	0.132
Kiganjo *R_bimp_*	8	1.02	0.327
Nakuru *R_bimp_*	5	1.72	0.157
Nairobi *R_bimp_*	7	1.07	0.323
Sagana *R_bimp_*	6	1.24	0.245

* Values of *p* designated by asterisks are statistically significant at *p* < 0.05 level under the Tukey–Kramer HSD test.

**Table 5:** Intercepts and slopes (±SEM) for regression equations fitting the models in Equations (1) and (2).

Model	Intercept ± SEM	*t* for Intercept	*p* > *t*	Slope ± SEM	*t* for Slope	*p* > *t*
aec*r_b_* = *T_c_*	0.326 ± 0.210	1.55	0.137	0.0132 ± 0.0039 *	3.34	0.004
imc*r_b_* = *T_c_*	0.078 ± 0.039	2.0	0.063	0.0016 ± 0.0007 *	2.13	0.047
imc*r_b_* = aec*r_b_*	0.070 ± 0.041	1.72	0.102	0.073 ± 0.033 *	2.16	0.044

* Statistically significant values of *t* (*p* < 0.05) for slopes are marked by asterisks.
